# Merkel Cell Carcinoma of the Head and Neck: A Single Institutional Experience

**DOI:** 10.1155/2013/325086

**Published:** 2013-01-10

**Authors:** G. Morand, D. Vital, T. Pézier, D. Holzmann, M. Roessle, A. Cozzio, G. F. Huber

**Affiliations:** ^1^Division of Otolaryngology, Head and Neck Surgery, University Hospital Zurich, Frauenklinikstraße 24, 8091 Zurich, Switzerland; ^2^Institute of Surgical Pathology, University Hospital Zurich, Frauenklinikstraße 24, 8091 Zurich, Switzerland; ^3^Division of Dermatology, University Hospital Zurich, Frauenklinikstraße 24, 8091 Zurich, Switzerland

## Abstract

Merkel cell carcinoma (MCC) is a rare cutaneous malignancy occurring mostly in older immunocompromized Caucasian males. A growing incidence of MCC has been reported in epidemiological studies. Treatment of MCC usually consists of surgical excision, pathological lymph node evaluation, and adjuvant radiotherapy. This paper reports the experience of a single tertiary center institution with 17 head and neck Merkel cell carcinoma patients. Median followup for the cohort was 37.5 months. After five years, recurrence-free survival, disease specific survival, and overall survival were 85%, 90%, and 83%, respectively. Our limited data support the use of adjuvant radiotherapy. We also report two cases of MCC located at the vestibule of the nose and two cases of spontaneous regression after diagnostic biopsy. About 40% of our patients were referred to our center for surgical revision and pathological lymph node evaluation. Increased awareness of MCC and an interdisciplinary approach are essential in the management of MCC.

## 1. Introduction

In 1875, Merkel, professor of anatomy at the University of Rostock, Germany, for the first time described “Tastzellen” (touch cells)—later known as Merkel cells—in the epidermis of domestic animals and humans [1]. In 1972, Toker first reported a case series of five patients with “trabecular carcinoma” and recognized a “distinct pathological entity,” with a “capricious clinical behaviour” [2]. It took Tang and Toker another six years to determine that trabecular carcinoma “most probably” derives from Merkel cells [3].

More recently, in 2008, a previously unknown polyomavirus was found to be integrated in Merkel cell carcinoma (MCC) [4]. Now known as Merkel Cell polyomavirus (MCV), this virus is indeed thought to be a causative agent in MCC [5, 6] and has been associated with about 80% of MCC cases [4, 7–10]. A previous study from our institution reported similar prevalence of MCV in MCC [11]. Conflicting evidence exists about the prognostic value of MCV status [7, 8, 12–14].

Important differential diagnoses of MCC are basal cell carcinoma, small cell melanoma, lymphoma, small blue round cell tumours, and especially metastatic small cell lung carcinoma [15]. A thorough histo- or cytopathological workup including immunohistochemistry (e.g., CK20, TTF-1, and neuroendocrine markers) [16] combined with entire examination and clinical history usually allows to differentiate the above mentioned entities.

A recent study by Smith et al. [17] drawn from an US-population based database (SEER) with over 4,300 MCC patients showed that 48% of all MCC are primarily located in the head and neck area (HN-MCC), with men representing about 61% of the patient population. The overwhelming majority of patients were Caucasian.

Hodgson [18] reported a incidence of Merkel cell carcinoma of 0.44/100,000/year in 2001 from the same database. Interestingly, there seems to be a sharp increase in the number of cases being reported. Hodgson performed a comparative analysis and showed an 8% estimated annual percentage change (EAPC) in MCC incidence from 1986 until 2001. This change was attributed to the ageing of population and the growing prevalence of immunocompromized patients, two known risk factors for MCC [18–22]. Furthermore, the authors noted that this change might also result from increased awareness and reporting of MCC [18]. A further risk factor for MCC is UVB exposure, as shown in epidemiological studies [23] and mutations analysis of TP53 [24]. Expression of p53 has been correlated to MCV-negative MCC [13, 14, 25], thus suggesting a different pathogenesis in MCV-positive and MCV-negative MCC.

The authors of another epidemiological study from The Netherlands [19] reported similar incidence trends of MCC with an EAPC of 8%. For the same period, the EAPC for melanoma and squamous cell carcinoma of the skin were 4% and about 2%, respectively.

The prognosis of MCC remains poor, with a 5-year relative survival for local, regional, and metastatic disease of about 75%, 50%, and 20%, respectively [19]. Smith et al. reported a 5-year disease specific survival for pN0, pN1, and M1 in HN-MCC patients of 83.3%, 58.3%, and 31.3%, respectively [17]. Epidemiological analyses have established several prognostic factors in HN-MCC [17]: male sex, >T2-primaries, N-positive, M-positive, and tumour location at the lip were shown to be independent negative risk factors. Unlike for nonhead and neck-MCC (NHN-MCC), increasing tumour size was not a prognostic factor for HN-MCC [17].

Lemos et al. have shown the critical impact of pathological lymph node evaluation for MCC patients [26]. For example, in the management of cutaneous melanoma, sentinel lymph node biopsy (SLNB) is now a standard of care [27]. Analogically, SLNB has gained popularity in the past years in the management of MCC, currently being recommended as standard treatment [28], independently of the size of the primary tumour [29–32], as SLNB permits a pathological lymph node evaluation with less morbidity compared to elective neck dissection [33, 34]. SLNB studies have shown that about 30% of cN0 MCC patients harbour occult metastasis [35, 36], with up to 20% false negative rate [37, 38]. 

Prophylactic irradiation is usually accepted as an alternative in the management of the clinically negative neck [39].

The superiority of adjuvant radiotherapy in local and regional control has been shown in a meta-analysis of observational studies [40]. A recent multicentric prospective randomized controlled trial conducted in France again showed improved locoregional control [41]. Both studies could not prove a significant advantage of adjuvant radiotherapy on disease specific survival.

For inoperable patients, an in-field control rate of 75% has been reported with radiotherapy alone, with 55 Gy as a minimum dose for macroscopic disease [42]. Other studies also show acceptable results for radiotherapy alone [43, 44].

The aim of this study was to report the experience of a single institution tertiary center in Switzerland.

## 2. Methods

After local ethics committee approval, we performed a retrospective chart review of all patients treated for a Merkel cell carcinoma in the department for Otorhinolaryngology—Head and Neck Surgery—at the University Hospital of Zurich, Switzerland, between January 1990 and August 2012. We searched our electronic patient database using the following keywords: “Merkel cell carcinoma,” “Merkel cell,” and the ICD-10-Code for Merkel cell carcinoma (C44.*). A single reviewer (GM) was responsible for sorting the eligible patients out of the generated list.

Inclusion criteria were confirmed histopathological diagnosis of Merkel cell carcinoma and location of the primary tumour in the head and neck. Only patients treated at our department were included. Patients treated by other departments (e.g., plastic surgery) were not included.

For each patient, the following parameters were assessed: tumour site, age, sex, immunosuppression, TNM-stadium (AJCC, 7th Edition 2010 [39]), treatment modality of local and regional disease, location of primary tumour, surgical margins, recurrence-free survival (RFS), disease specific survival (DSS), and overall survival (OS).

Descriptive statistics were performed using Microsoft Excel 2007. Kaplan Meier estimates were calculated using IBM SPSS 19 and the survival functions were compared by log rank tests.

## 3. Results

### 3.1. Demographics, Tumour Location, Staging, and Risk Factors

Seventeen patients (4 male, 13 female, ratio 1 : 3.3) met the inclusion criteria (Table 1). The mean age was 71 y (SD 15.8, median 73, range 40–89). Ten primaries (58%) were located on the cheek, two on the external ear (12%), two in the vestibule of the nose (12%), one at the lower lip (6%), one at the eyelid (6%), and one at the glabella (6%). Diabetes was reported for one patient (6%); HIV/AIDS or immunosuppression after organ transplant was not reported for any patients.

According to AJCC, 7th Edition 2010 [45], stage Ia was present in four cases (24%); stage Ib in one (6%); stage IIa in two (12%); stage IIb in two (12%); stages IIc and IIIa in zero; stage IIIb in four (24%); and stage IV in zero cases. In four patients (24%), tumour stage was not available because of missing data.

**Figure 1:**
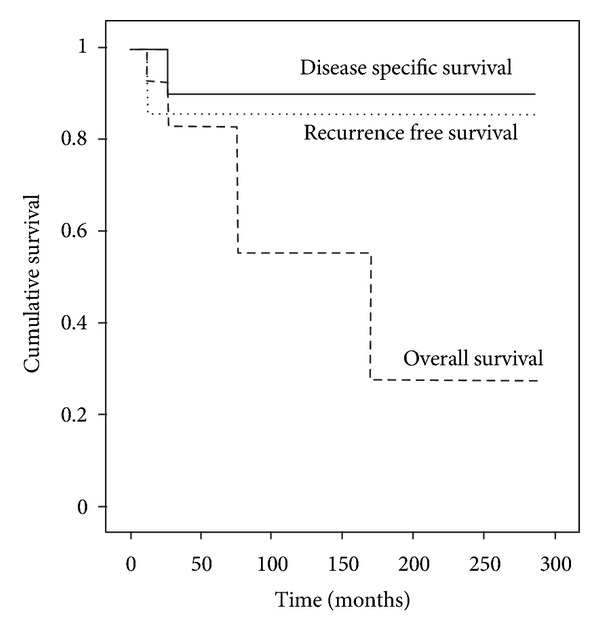
Survival analysis.

Median followup for the cohort was 37.5 months (mean 62, SD 72, range 5–288). One patient (6%) died of Merkel cell carcinoma, three (18%) of other causes. After five years, recurrence-free survival (RFS), disease specific survival (DSS), and overall survival (OS) were 85%, 90%, and 83%, respectively (Figure 1).

### 3.2. Treatment Options, Surgical Margins, Pathological Lymph Node Evaluation

Sixteen patients (94%) underwent primary surgical resection. One patient (6%) underwent primary radiotherapy, as surgical excision would have resulted in complete removal of the nose, which was unacceptable to the patient. This patient was excluded from further analysis.

In three out of sixteen patients (19%), surgical excision margins of 2 cm were reported and in two cases margins of 1 cm (13%). In one case (6%), Mohs surgery was used. 

In three cases (19%), margins smaller than 1 cm were used, twice because the surgeon did not consider MCC in the clinical differential diagnosis. For the remaining case, a two-step surgery was chosen, with the use of artificial skin graft, whilst a definitive histological margin was performed which led to a further excision a week later.

In seven cases (44%), surgical margins were not available; all these patients were referred to our institution after having primary surgery elsewhere.

All of the patients who had planned 2 cm excision margins had clear histological margins. One of the two patients with a planned 1 cm margin had to undergo a second operation to obtain clear histological margins. This patient (number 8) did not receive adjuvant radiotherapy as the primary was located at the lower lid and concerns were raised about the irradiation of the eye. He suffered a local recurrence one year later and had to undergo extended resection with orbital exenteration, free flap reconstruction, and adjuvant radiotherapy.

Of the patients who were initially operated in other hospitals and for those with planned excision margins of <1 cm, nine out of ten (90%) had to undergo at least one second surgical intervention. Data is incomplete for the last patient.

There was not statistical relation between surgical margins and recurrence-free survival (*P* = 0.47).

Of the sixteen patients who underwent primary surgical excision, sentinel lymph node biopsy was performed in five patients (31%) and was always negative. Mean number of sentinel nodes was 2.2 (range 1–4). 

Two patients (12%) underwent therapeutic selective neck dissection for clinically positive neck disease (patients 11 and 13).

Five patients (31%) had elective selective neck dissections, showing regional disease in two cases (12%). 

Five patients (31%) underwent, in addition to neck dissection, superficial parotidectomy, due to the localisation of the primary tumour and the expected lymphatic drainage. No disease was found in any of the parotid specimens.

Five patients (31%) did not have any pathological node evaluation, in one case because of the patient comorbidities. This patient (number 4) went on to develop regional recurrence 11 months later. He could not undergo salvage neck dissection and was treated with locoregional radiotherapy. A few months later, he had distant metastatic disease and died twenty-six months after the initial surgery.

### 3.3. Recurrence, Radiotherapy, and Outcome

Two patients (12%) developed the locoregional recurrence. Patient 8 had a primary at the lower eyelid with a local recurrence 12 months after primary surgery. Patient 4 showed a regional recurrence, as discussed above. These patients received radiotherapy after locoregional recurrence.

In eleven patients (69%), adjuvant radiotherapy was recommended. One patient (6%) refused and another patient could not undergo radiotherapy because of severe wound healing problems.

Three patients (31%) did not have any radiation. Two of them underwent excision biopsy outside our clinic and were referred for revision surgery and pathological node evaluation. For both patients, the revision specimens and SLNB were free of tumour in the histopathological analysis. Adjuvant radiotherapy was therefore not performed.

Of the nine patients who had adjuvant radiotherapy following surgery, none suffered recurrence. Without adjuvant radiotherapy following surgery, two of six patients (33%) developed locoregional recurrence. There was a trend to a better RFS in patients who underwent adjuvant radiation (*P* = 0.051, log rank test).

Mean adjuvant radiation dose was 62.4 Gy (SD 4.8, median 62, range 54–70).

## 4. Discussion

MCC is a rare malignancy, occurring mostly in older immunocompromized Caucasian males. As the population in most Western countries continues ageing [46] and the prevalence of immunosuppression increases [47], epidemiological studies have shown an increase in MCC incidence.

The growing incidence of MCC has led to an increased awareness and reporting of MCC. Searching for “Merkel cell carcinoma” in PubMed database reveals a steady increase in results by year, with about 40 publications per year in the 90s, to over 160 publications for the year 2010 or 2011.

We report here a single institution retrospective analysis. The demographics of our patient cohort differ from other retrospective reports, as do our survival rates [17, 48–51]. The mean age is slightly lower than in other larger reports [17] and we have a predominance of women and no immunosuppressed patients, which could be explained by our relatively small patient cohort.

Considering a cohort with elderly patients, a difference between the OS and the DSS can be explained by comorbidities. We had in fact more disease unrelated deaths as disease specific deaths in the follow-up period.

In comparison to other reports [26, 40, 52], our favourable DSS and DFS could be explained by high rate of combined therapy, high rate of pathological lymph node dissection, low rate of distant disease at diagnosis, a predominance of women [17], and lack of immunosuppressed patients [22]. Our results must however been interpreted in light of the small cohort size.

As reported elsewhere [53], we did not find surgical margins to significantly affect recurrence-free survival. This is consistent with the fact that clear histological margins were obtained for every patient, independently of the surgical excision margins chosen initially. For HN-MCC, we recommend margins of 1 cm, or nonfeasible, two-step or Mohs surgery [28, 54, 55].

We observed a trend supporting the use of adjuvant radiotherapy. Although not significant, this result complies with stronger evidence [40, 41].

We report two cases of MCC occurring in the vestibule of the nose. This location has been reported very rarely in the literature [56]. One patient was treated using Mohs surgery. For the other patient, primary radiotherapy was chosen because an ablation of the nose would have been necessary to obtain clear surgical margins.

We also report two cases of surgical revision after external excision biopsy with lack of MCC cells in the revision specimen and SLNB, thus who either had been fully excised surgically or with spontaneous regression following biopsy, as already described in many other reports [56–61].

Analogically, MCC of unknown primary has also been described [62, 63]. Although the mechanism of regression is not known, immune infiltration has been proposed [64]. However, in a recent study with 37 patients, no significant increase in intratumoral CD8 T-cell infiltration after biopsy could be found [65].

As seven out of seventeen of the patients (41%) in this study were referred to surgical revision and pathological lymph node evaluation, we think that continuing education is essential, particularly for house physicians and general practitioners, who need to be aware of this rare but increasing malignancy, usually gentle in presentation [66, 67].

Our results should be interpreted cautiously, as biased by missing data and heterogeneous population. Our statistical analysis lacks power due to our low number of patientS and events. As a tertiary center, we also suffer referral bias. Nevertheless, bearing these caveats in mind, we believe that a few lessons can be learned from this paper.

In conclusion, we believe that increased awareness of MCC is essential to ensure an optimal initial management. Failure to do so can lead to a higher number of surgical interventions and missing or incomplete pathological staging.

Surgical removal of HN-MCC should assure oncological sufficient treatment, while preserving cosmetic or functional essential structure. An alternate surgical technique should be used, as appropriate.

To overcome these challenges, we strongly believe that a multidisciplinary approach and collaboration is essential. In accordance with the available literature, in Zurich, we have established an internal guideline, with a systematic assessing of several variables, thus allowing high quality data for further studies.

## Figures and Tables

**Table 1:** Summary of patients characteristics.

°	Age	Sex	Location	Side	T	N	M	Therapy	Recurrence	Followup	Outcome
1	73	f	Cheek	Right	pT2	cN0	M0	Excision only	No	170	MCC unrelated death
2	81	f	Cheek	Left	pT2	pN0 (0/31)	M0	Excision + ND + RT	No	9	RFS
3	52	f	Vestibule of nose	Right	pT1	pN0 (sn 0/4)	M0	Excision + SLNB	No	18	RFS
4	88	f	Glabella	Middle	pT1	cN0	M0	Excision only	Yes^§^	27	disease specific death
5	86	f	Pinna	left	pTx	cN0	M0	Excision + RT*	No	0	lost to followup
6	64	m	Cheek	Right	pT1	pN0 (sn 0/1)	M0	Excision + SLNB + RT	No	72	RFS
7	57	f	Cheek	Right	pT1	pN0 (sn 0/3)	M0	Excision + SLNB	No	66	RFS
8	73	f	Lower lid	Left	pTx	pN0 (sn 0/1)	M0	Excision + SLNB	Yes^*♮*^	74	MCC unrelated death
9	86	m	Cheek	Left	pT2	pN0 (0/23)	M0	Excision + ND + RT**	No	36	RFS
10	65	f	Cheek	Left	pTx	pN0 (sn 0/2)	M0	Excision + SLNB + RT	No	39	RFS
11	40	f	Cheek	Left	pT1	pN1b (4/26)	M0	Excision + ND + RT	No	21	RFS
12	62	f	Cheek	Right	pT1	pN0 (0/40)	M0	Excision + ND + RT	No	24	RFS
13	86	m	Pinna	Right	pT1	pN2 (3/21)	M0	Excision + ND + RT	No	11	MCC unrelated death
14	51	f	Cheek	Right	pTx	cN0	M0	Excision + RT	No	288	RFS
15	72	m	Lower lip	Middle	pTx	pN1b (3/28)^$^	M0	Excision + ND + RT	No	63	RFS
16	77	f	Cheek	Right	pT2	pN1b (1/25)	M0	Excision + ND + RT	No	72	RFS
17	89	f	Vestibule of nose	Left	cT2	cN0	M0	Radiotherapy only	No	5	RFS

Age in years; f: female; m: male. TNM stadium according to AJCC, 7th Edition, 2010. RT: radiotherapy. ^$^bilateral neck dissection, left 3/14, right 0/14. *RT refused by patient. **RT could not be performed because of wound healing problems. ^§^regional recurrence after 11 months. ^*♮*^local recurrence after 12 months. Followup in months. SLNB: sentinel lymph node biopsy. ND: neck dissection. RFS: recurrence-free survival. MCC: Merkel cell carcinoma.
